# Transfer of motor skill between virtual reality viewed using a head-mounted display and conventional screen environments

**DOI:** 10.1186/s12984-020-00678-2

**Published:** 2020-04-10

**Authors:** Julia M. Juliano, Sook-Lei Liew

**Affiliations:** 1 Neural Plasticity and Neurorehabilitation Laboratory, Neuroscience Graduate Program, University of Southern California, Los Angeles, CA USA; 2 Neural Plasticity and Neurorehabilitation Laboratory, Division of Occupational Science and Occupational Therapy, University of Southern California, Los Angeles, CA USA; 3 USC Stevens Neuroimaging and Informatics Institute, Department of Neurology, University of Southern California, Los Angeles, CA USA

**Keywords:** Virtual reality, Head-mounted display, Motor skill acquisition, Transfer, Presence

## Abstract

**Background:**

Virtual reality viewed using a head-mounted display (HMD-VR) has the potential to be a useful tool for motor learning and rehabilitation. However, when developing tools for these purposes, it is important to design applications that will effectively transfer to the real world. Therefore, it is essential to understand whether motor skills transfer between HMD-VR and conventional screen-based environments and what factors predict transfer.

**Methods:**

We randomized 70 healthy participants into two groups. Both groups trained on a well-established measure of motor skill acquisition, the Sequential Visual Isometric Pinch Task (SVIPT), either in HMD-VR or in a conventional environment (i.e., computer screen). We then tested whether the motor skills transferred from HMD-VR to the computer screen, and vice versa. After the completion of the experiment, participants responded to questions relating to their presence in their respective training environment, age, gender, video game use, and previous HMD-VR experience. Using multivariate and univariate linear regression, we then examined whether any personal factors from the questionnaires predicted individual differences in motor skill transfer between environments.

**Results:**

Our results suggest that motor skill acquisition of this task occurs at the same rate in both HMD-VR and conventional screen environments. However, the motor skills acquired in HMD-VR did not transfer to the screen environment. While this decrease in motor skill performance when moving to the screen environment was not significantly predicted by self-reported factors, there were trends for correlations with presence and previous HMD-VR experience. Conversely, motor skills acquired in a conventional screen environment not only transferred but improved in HMD-VR, and this increase in motor skill performance could be predicted by self-reported factors of presence, gender, age and video game use.

**Conclusions:**

These findings suggest that personal factors may predict who is likely to have better transfer of motor skill to and from HMD-VR. Future work should examine whether these and other predictors (i.e., additional personal factors such as immersive tendencies and task-specific factors such as fidelity or feedback) also apply to motor skill transfer from HMD-VR to more dynamic physical environments.

## Background

The use of virtual reality (VR) in rehabilitation has been growing exponentially over recent years [1, 2]. Clinical applications of VR have been shown to be engaging and motivating [3, 4] with promising results suggesting VR interventions are comparable [5] or in some cases superior [6, 7] to conventional rehabilitation. However, while a number of studies have reported benefits of using VR for cognitive and motor rehabilitation, there are also reports on the limitations of using these devices for clinical applications [8, 9]. In particular, some studies have shown that VR interventions are not effective at improving motor performance in the real world due to a lack of motor skill transfer (i.e., the application of a motor skill in a novel task or environment [10]) [11, 12].

Concerns about motor skill transfer from virtual to real environments are even greater when specifically considering the use of VR viewed using a head-mounted display (HMD-VR). HMD-VR provides a more immersive experience compared to conventional environments (e.g., computer screens) and results in increased levels of presence (i.e., the illusion of actually being present in the virtual environment) and embodiment (i.e., the perceptual ownership of a virtual body in a virtual space) [13, 14] that modulate behavior [15] and impact performance on motor learning and rehabilitation applications (e.g., gait, balance, neurofeedback tasks) [16–18]. Additionally, motor learning in HMD-VR (e.g., upper extremity visuomotor adaptation) has been shown to rely on different learning processes compared to a conventional screen environment [19]. Given the differences in immersive experiences and learning processes between HMD-VR and conventional environments, it can be assumed that individuals may experience these environments as separate contexts. Studies have found the context of the training environment to affect the transfer of motor skills [20], where motor performance may decrease when testing occurs in an environment different from training [21]. However, only a small number of studies have specifically explored motor skill transfer of from an HMD-VR training environment to a more conventional environment (e.g., computer screen or real world) [22–26]. Among these studies, there are again conflicting results, with some studies finding successful motor skill transfer from HMD-VR to the real world [22, 23], and others not [24–26].

There is also large interindividual variability within the results, and this variability suggests there may be particular tasks or particular individuals that will be more successful in transferring HMD-VR motor skills to the real world. Understanding the task-related or personal factors that mediate learning and transfer from HMD-VR environments should be examined in order to understand what makes HMD-VR interventions effective. One advantage of HMD-VR over conventional screen environments is the ability to realistically simulate the real world which allows for greater task specificity [27]. Task-related factors such as fidelity (i.e., imitation of the real environment) and dimensionality (i.e., matching dimensions between virtual and real environments) between HMD-VR and the real world have been shown to influence lower extremity motor performance [28] and have been suggested to have an influence on transfer in both lower and upper extremity motor transfer [29, 30]. Individual differences in personal factors such as gender, age, video game experience, prior technical computer literacy, and computer efficacy seemed to influence transfer from HMD-VR to the real world in studies examining the transfer of spatial knowledge acquired in an HMD-VR environment [26, 31]. However, the individual differences on both task-related and personal factors have not been extensively examined in HMD-VR motor skill transfer. We begin to address this gap by examining whether individual personal factors facilitate better transfer from upper extremity motor skill acquisition in HMD-VR to a conventional screen environment.

In the current study, we examined: (1) whether transfer of upper extremity motor skills occurs between HMD-VR and conventional screen environments, and (2) what personal factors predict transfer between environments. Given the variability of motor skill learning and transfer in previous studies [22–26, 29], we hypothesized that individual motor performance would vary after transfer to a novel environment, and that this variability could be predicted by individual differences in variables such as presence in the training environment, prior experience with HMD-VR, or non-VR video games.

## Methods and materials

### Participants

Seventy-four healthy adults were recruited. Participants were randomized into two groups (Train-HMD-VR, Train-Screen). Three participants in the Train-Screen group were excluded from the analysis as a result of performing all trials in the Baseline training block incorrectly (see *Analyses*) and one participant in the Train-HMD-VR group was excluded from the analysis as a result of being an outlier, which was defined as being beyond three standard deviations from the group mean motor skill in at least one of the blocks. This resulted in a total of seventy participants (53 females/16 males/1 other, aged: M = 25.81, SD = 4.71) with thirty-five participants in each group included in the analysis. A statistical power analysis was performed for sample size estimation based on data from a pilot study of this work (*N* = 12) [32]. The effect size in this study was d = 0.38. With an alpha = 0.05 and power = 0.60, the projected sample size need with this effect size was approximately *N* = 35. Eligibility criteria included healthy, self-reported right-handed individuals and no previous experience with the motor skill task (see *Experimental design*). Written informed consent was obtained from all subjects. The experimental protocol was approved by the University of Southern California Institutional Review Board and performed in accordance with the 1964 Declaration of Helsinki.

### Experimental design

**Fig. 1:**
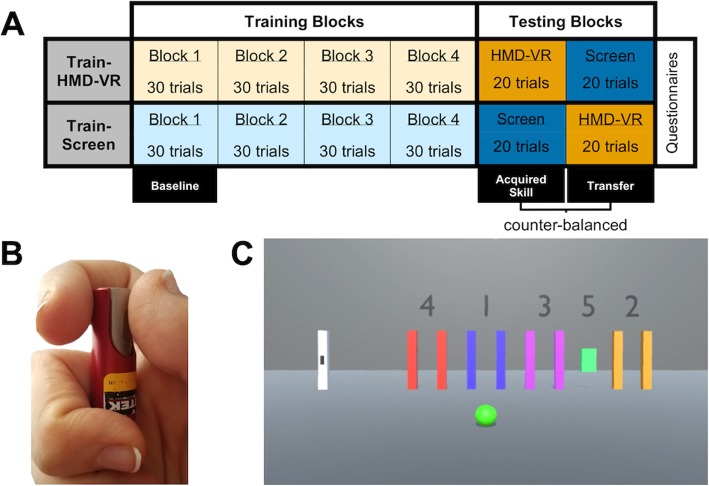
Experimental paradigm. **a** Experimental design. **b** Pinch force between the thumb and index finger was applied to a small force transducer to move the cursor in the SVIPT. **c** Sequential Visual Isometric Pinch Task (SVIPT) display. Participants were asked to apply force to the force transducer, which translated into the movement of a small black cursor (shown at the home position in the white bar) moving horizontally to the right in the environment. The cursor moved left by reducing force. Instructions were to move the cursor between the gates, in order from 1 to 5, as quickly and accurately as possible, without over- or under-shooting any of the gates

Figure 1a provides an overview of the experimental design. The experiment consisted of training and testing blocks in which participants completed a modified version of the Sequential Visual Isometric Pinch Task (SVIPT) [33]. In this task, participants were instructed to apply varying degrees of isometric force between their thumb and index finger to a small pinch force sensor (Futek Pinch Sensor FSH01465; Futek IPM FSH03633; Fig. 1b) to move a cursor between numbered colored gates as quickly and accurately as possible (Fig. 1c). A small circle at the bottom of the screen changed from red to green to indicate the start of each trial. For each trial, no time limit was given and trial completion time was recorded. At the end of each trial, the small circle at the bottom of the screen changed from green to red and participants received auditory feedback (a pleasant “ding” if the cursor correctly entered all the gates or an unpleasant “buzz” if the cursor missed one or more of the gates). A two-second time interval was given between each trial.

**Fig. 2:**
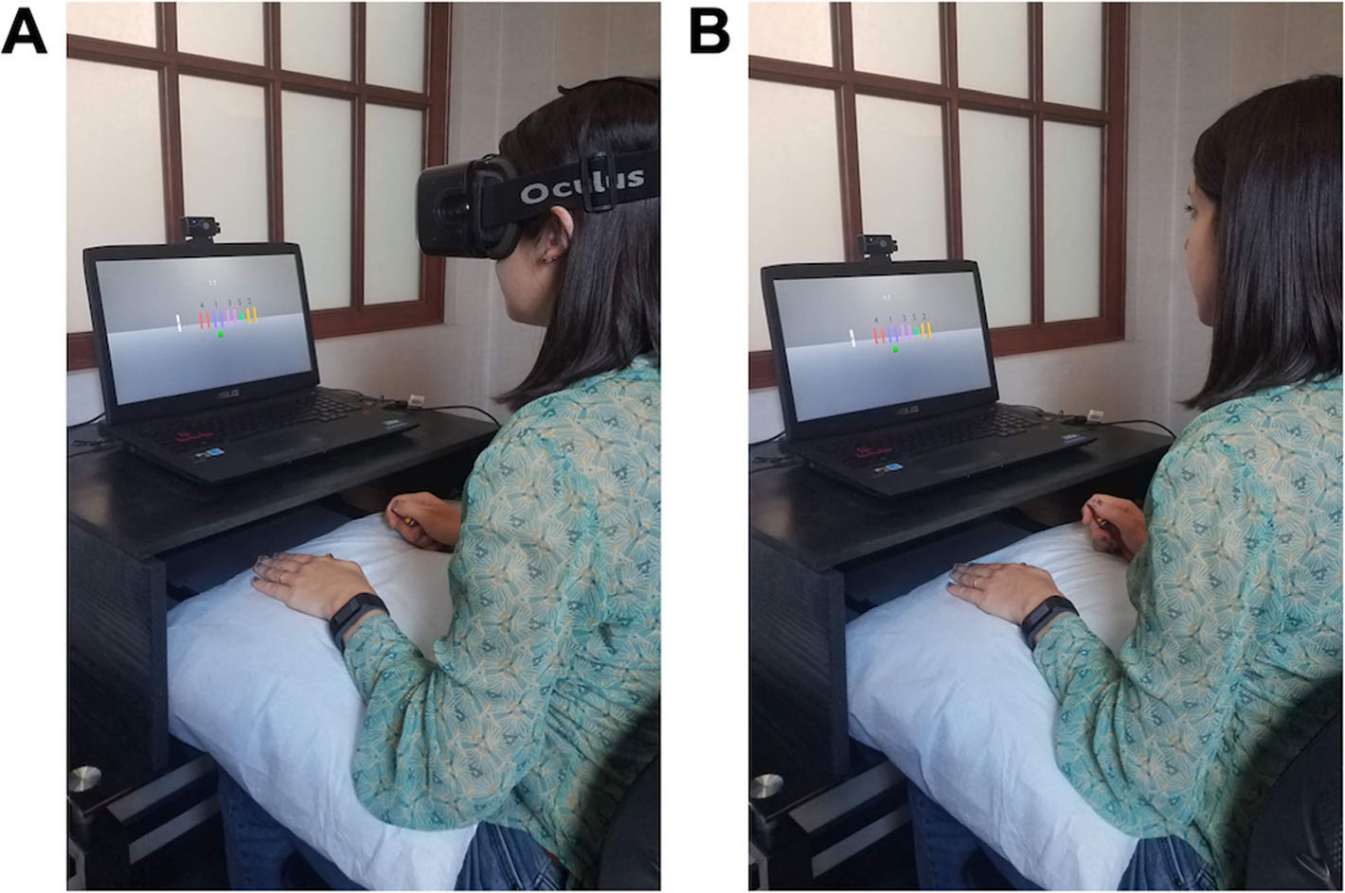
Training and testing environments. Participants were trained on a motor skill task in either the HMD-VR or Screen environment and were then tested on the same task in both environments. **a** HMD-VR environment; the stimulus shown in the HMD-VR display is also shown on the computer screen. **b** Screen environment

Participants completed 4 training blocks (Training Blocks 1–4) consisting of 30 SVIPT trials either in an HMD-VR (Fig. 2a; Train-HMD-VR) environment or on a computer screen (Fig. 2b; Train-Screen). Block 1 was considered the Baseline training block for each group. After completion of the training blocks, all participants completed 2 counter-balanced testing blocks consisting of 20 SVIPT trials in an HMD-VR environment and on a computer screen. We defined the testing block that matched the training condition as the “Acquired Skill” testing block (e.g., train in HMD-VR, test in HMD-VR). There was no difference in either group between the last block of training (Block 4) and the Acquired Skill testing block; thus, the Acquired Skill testing block was used as a proxy for total amount of motor skill within the assigned training environment. We defined the testing block that was different from the training condition as the “Transfer” testing block (e.g., train in HMD-VR, test in Screen). Importantly, there was no difference in the order of testing block completion for either group. Lastly, after completion of both training and testing blocks, participants were asked to complete three questionnaires (see *Questionnaires*).

### Training and testing environments

The environments for all blocks were designed using the game engine development tool, Unity 3D (Version 5.6.6). In the blocks where participants were in the HMD-VR environment, participants performed the task in a head-mounted display (Oculus Rift DK2). In the blocks where participants were in the Screen environment, participants performed the task on a 17.3 in., 1920 × 1080 pixel resolution computer laptop (ASUS ROG G751JY-DH71). The HMD-VR environment was created based on a fixed coordinate system that did not depend on the participant’s head position. All participants were physically seated in the same location for all blocks and used the same force transducer for the task. The only difference between HMD-VR and Screen blocks is that participants put on the HMD-VR headset for HMD-VR blocks.

### Questionnaires

Participants were asked to complete two Likert-scale questionnaires regarding their reaction to the training environment; the first questionnaire related to participants’ simulator sickness and the second questionnaire related to participants’ level of presence. The first questionnaire was the simulator sickness questionnaire, adapted from Kennedy, Lane, Berbaum, & Lilienthal (1993) [34], and consisted of a series of questions to gauge participant sickness level and was given both before and after the task. Questions were collapsed along four main themes: nausea, oculomotor reactions, disorientation, and overall simulator sickness. The second questionnaire was the presence questionnaire, which was adapted from Witmer & Singer (1998) [35] and revised by the UQO Cyberpsychology Lab (2004). It consisted of a series of questions to gauge the participant’s sense of presence in the training environment. Questions were measured along five main themes: realism, possibility to act, quality of interface, possibility to examine, and self-evaluation of performance. Participants were also asked questions regarding their gender, age, whether or not they played video games, and whether or not they had previous experience using HMD-VR. Both these questions and the presence questionnaire were administered at the end of the experiment.

### Analyses

All analyses were complete in R (Version 3.5.3) using R Studio (Version 1.1.423). We assessed the normality of each variable using skewness and kurtosis of the distribution. Given our sample size, we considered a variable with an absolute skewness or kurtosis value of less than 1.96 as normally distributed [36]. Only simulator sickness variables were considered non-normally distributed (see *Simulator sickness, presence, and subjective measures*). Significance was defined as *p* < 0.05 unless corrected with multiple comparisons using a Bonferroni correction; these corrections are specified for each analysis.

#### Simulator sickness, presence, and subjective measures

To examine any differences between groups in the variables calculated based on simulator sickness questionnaire responses (i.e., nausea, oculomotor reactions, disorientation, and overall simulator sickness), we use a Mann-Whitney U test as either skewness or kurtosis values were greater than 1.96 for each variable. To examine any differences between groups in the variables calculated based on presence and subjective measures questionnaire responses, we used an unpaired t-test for quantitative variables (i.e., realism, possibility to act, quality of interface, possibility to examine, self-evaluation of performance, age) and a chi-squared test for qualitative variables (i.e., gender, video game use, and previous HMD-VR experience). Significance was defined as *p* < 0.0125 for the four simulator sickness variables and defined as *p* < 0.0055 for the nine presence and subjective variables.

#### Motor skill acquisition and motor skill transfer

Motor skill was calculated based on a formula first presented in Reis et al. 2009 [33], which measures the ratio of speed to accuracy over the trials in each block. Motor skill for each block is calculated as:

$$ \mathrm{Motor}\kern0.34em \mathrm{Skill}=\ln \kern0.17em \left(\frac{1-\mathrm{error}\kern0.34em \mathrm{rate}}{\mathrm{error}\kern0.34em \mathrm{rate}\left(\ln \kern0.17em {\left(\mathrm{duration}\right)}^b\right)}\right), $$

where error rate and duration are averaged over trials in each block and *b* is a free parameter that equals 5.424 [33]. If error rate = 0 for a given block (i.e., all trials were incorrect), the resulting motor skill calculation would be undefined. For each group, we assessed motor skill acquisition and motor skill transfer; *motor skill acquisition* is the increased performance in the trained environment and *motor skill transfer* is the maintained performance in the untrained environment after training. To calculate motor skill acquisition, we compared motor skill from the Baseline training block to the Acquired Skill testing block for each group. Additionally, we compared the motor skill on the Baseline training block to the Transfer testing block to assess whether training in one enviorment had an effect on motor skill performance in the other environment. To calculate motor skill transfer we compared the Acquired Skill testing block to the Transfer testing block for each group. A paired t-test was used for these within-group comparisons with significance defined as *p* < 0.025 since comparisons were made for each group. Lastly, to quantify individual motor skill transfer, we took the difference in motor skill between the Transfer testing block and the Acquired Skill testing block across individuals and compared overall motor skill transfer between the two groups; an unpaired t-test was used for this between group comparison.

#### Identifying individual factors that predict motor skill transfer

Finally, to examine which individual factors predicted motor skill transfer, we considered the nine presence and subjective variables in a multivariate linear regression model (i.e., realism, possibility to act, quality of interface, possibility to examine, self-evaluation of performance, age, gender, video game use, and previous HMD-VR experience) for each group. To identify variables that strongly predicted motor skill transfer, we used the regularization technique lasso [37] with 10-fold cross-validation, which shrinks some coefficients and sets others to zero. Shrinking coefficient estimates through lasso can reduce the variance at the cost of a small increase in bias [38] and has been suggested for datasets with a similar sample size to predictor ratio [39, 40]. Specifically, we trained a lasso model with cross-validation on 75% of the dataset using the glmnet R function [41]. Then, using the tuning parameter lambda that produced the minimum mean square error (MSE), we calculated the prediction error on the remaining 25% of the dataset and refit the lasso model using the full dataset. This resulted in a sparse linear model that is more interpretable and only includes a subset of the variables included in the initial linear model. Variance inflation factor (VIF) was calculated for each predictor to check for multicollinearity; we considered a VIF value less than 3.3 as meeting the assumption of collinearity [42]. For exploratory purposes, we then individually examined the variables in a univariate linear regression model to determine whether any variables on their own could explain motor skill transfer. Qualitative predictors (i.e., gender, video game use, and previous HMD-VR experience) in both multivariate and univariate linear regression models were encoded as dummy variables.

## Results

### No differences in simulator sickness, presence, and subjective measures between environments

To assess differences in simulator sickness level between training environments, we compared scores of nausea, oculomotor reactions, disorientation, and overall simulator sickness between groups and found no significant difference for each of the measures (Supplementary Table 1), suggesting that this type of motor skill training does not produce any additional simulator sickness side effects in HMD-VR compared to what is experienced when training on a computer screen. Additionally, we compared each of the nine prediction variables (realism, possibility to act, quality of interface, possibility to examine, self-evaluation of performance, age, gender, video game use, and previous HMD-VR experience) and found no significant differences between the two groups (Supplementary Table 2).

### No differences in motor skill acquisition between training environments

To assess initial and end of training performance between training environments, we compared motor skill between groups at the Baseline training block (Block 1) and the Last training block (Block 4). There were no differences between groups in Baseline training blocks (t (68.0) = 0.44, *p* = 0.6626; Train-HMD-VR: M = − 4.83, SD = 1.12; Train-Screen: M = − 4.95, SD = 1.13) and in the Last training blocks (t (66.3) = 0.26, *p* = 0.7941; Train-HMD-VR: M = − 2.93, SD = 1.06; Train-Screen: M = − 3.00, SD = 0.90). To compute individual participant acquisition rates, we applied a linear-log linear regression to motor skill across the four training blocks [43]. We found similar acquisition rates (i.e., slopes from regression model) between the two groups (t (58.8) = − 0.41, *p* = 0.6839; Train-HMD-VR: M = 1.36, SD = 0.84; Train-Screen: M = 1.43, SD = 0.56), suggesting that motor skill acquisition occurred at a similar rate across HMD-VR and Screen groups.

### Motor skill acquisition and motor skill transfer

#### Motor skill acquisition occurs in both environments

**Fig. 3:**
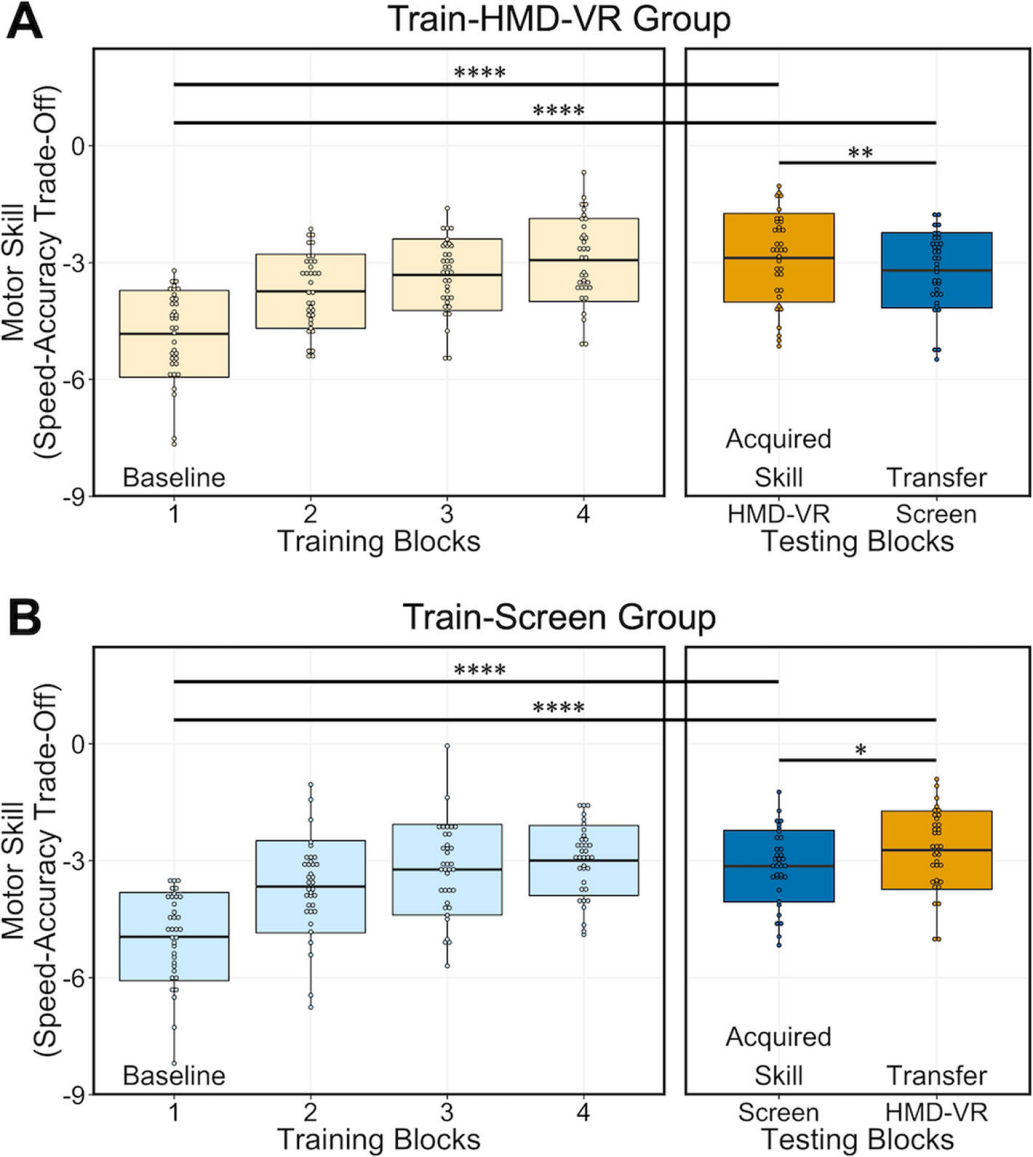
Motor skill shown for the Train-HMD-VR group in (**a**) and the Train-Screen group in (**b**). Light yellow blocks are HMD-VR training blocks, dark yellow blocks are HMD-VR testing blocks. Light blue blocks are Screen training blocks, dark blue blocks are Screen testing blocks. **a** Motor skill across training blocks in Train-HMD-VR group and both corresponding testing blocks. Participants increased their motor skill after training in HMD-VR (t(34) = − 11.42, *p* < 0.0001). Transfer to a computer screen occurred as a result of HMD-VR training (t(34) = − 9.12; p < 0.0001); however, the motor skills transferred to a computer screen was less than the motor skills in HMD-VR (t(34) = 2.83, *p* = 0.0078). **b** Motor skill across training blocks in Train-Screen group and both corresponding testing blocks. Participants increased their motor skill after training on a computer screen (t(34) = − 9.68, p < 0.0001). Transfer to HMD-VR occurred as a result of computer screen training (t(34) = − 12.52; p < 0.0001); however, the motor skills transferred to HMD-VR was greater than the motor skill on a computer screen (t(34) = − 2.59, *p* = 0.0142). Indicators of significance: *p* < 0.05^*^, *p* < 0.01^**^, p < 0.0001^****^

To ensure that motor skill acquisition occurred in both environments, we compared the motor skill between the Baseline training block and the Acquired Skill testing block for the Train-HMD-VR group and for the Train-Screen group separately. On average, we found motor skill acquisition occurred after training in HMD-VR (Train-HMD-VR: t (34) = − 11.42, *p* < 0.0001; Baseline (Block 1): M = − 4.83, SD = 1.12, Acquired Skill (HMD-VR): M = − 2.88, SD = 1.14; Fig. 3a) and after training on a computer screen (Train-Screen: t (34) = − 9.68, p < 0.0001; Baseline (Block 1): M = − 4.95, SD = 1.13, Acquired Skill (Screen): M = − 3.14, SD = 0.92; Fig. 3b). This suggests that motor skill acquisition on an isometric pinch force task can occur both in HMD-VR as well as on a more conventional screen environment.

To assess whether training in HMD-VR had an effect on motor skill performance on a computer screen, we compared the motor skills on the Baseline training block to the Transfer testing block for the Train-HMD-VR group. We found a significant difference in performance between the HMD-VR Baseline training block (M = − 4.83, SD = 1.12) and the computer screen Transfer testing block (M = − 3.20, SD = 0.96; t (34) = − 9.12; *p* < 0.0001; Fig. 3a), suggesting that motor skill training in HMD-VR increased the motor skill performance on a computer screen, compared to if no HMD-VR training occurred. To assess whether training on a computer screen had an effect on motor skill performance in HMD-VR, we compared the motor skills on the Baseline training block to the Transfer testing block for the Train-Screen group. We found a significant difference in performance between the computer screen Baseline training block (M = − 4.95, SD = 1.13) and the HMD-VR Transfer testing block (M = − 2.73, SD = 1.01; t(34) = − 12.52; *p* < 0.0001; Fig. 3b), suggesting that motor skill training on a computer screen increased the motor skill performance in HMD-VR, compared to if no training on a computer screen occurred.

#### Motor skill transfer to computer screen: performance decreases

To assess motor skill transfer to a computer screen after training in HMD-VR, we compared the Acquired Skill testing block (HMD-VR) to the Transfer testing block (Screen) and found a significant difference (t(34) = 2.83, *p* = 0.0078; Fig. 3a), where motor skill was lower in the Transfer testing block (M = − 3.20, SD = 0.96) compared to the Acquired Skill testing block (M = − 2.88, SD = 1.14). This suggests that performance decreased after transfer to the untrained computer screen environment. As a reminder, there was no significant difference in motor skill based on the order of the Acquired Skill and Transfer blocks, which were counterbalanced across individuals.

#### Motor skill transfer to HMD-VR: performance increases

We then assessed motor skill transfer to HMD-VR after training on a computer screen. To examine this, we compared the Acquired Skill testing block (Screen) to the Transfer testing block (HMD-VR) and found a significant difference (t(34) = − 2.59, *p* = 0.0142; Fig. 3b), where motor skill was higher in the Transfer testing block (M = − 2.73, SD = 1.01) compared to the Acquired Skill testing block (M = − 3.14, SD = 0.92). This suggests that performance increased after transfer to the untrained HMD-VR environment. In this group, there was also no significant difference in motor skill based on the order of the Acquired Skill and Transfer blocks, which were counterbalanced across individuals.

#### Individual motor skill transfer

**Fig. 4:**
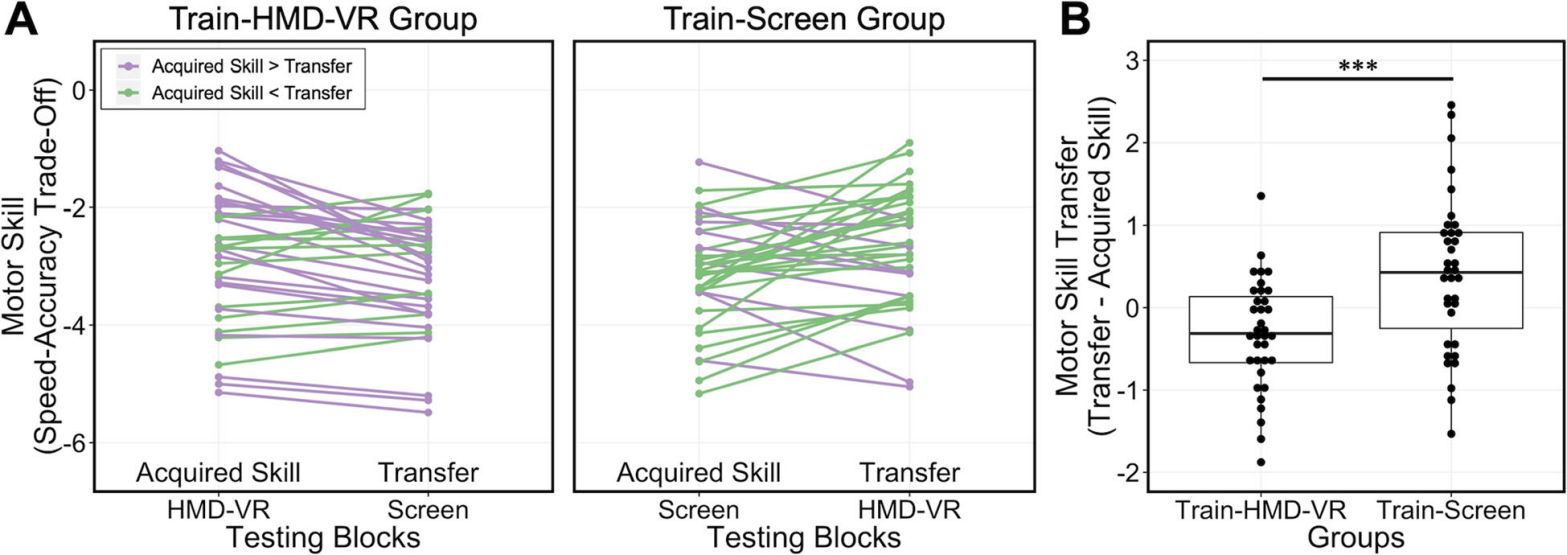
**a** Individual motor skill differences on Acquired Skill and Transfer testing blocks for both the Train-HMD-VR group (left) and the Train-Screen group (right). Purple represents individuals with greater motor skill on the Acquired Skill testing block and green represents individuals with greater motor skill on the Transfer testing block. **b** The y-axis shows “Motor Skill Transfer”, which is defined as the motor skill on the Transfer block minus the motor skill on the Acquired Skill block for each individual. There was a significant difference in average motor skill transfer between the Train-HMD-VR group (left) and Train-Screen group (right; t(61.5) = 3.75, *p* = 0.0004). Dots represent individuals, the box represents the first and third quartiles, and the line represents the median. *p* < 0.001^***^

In the Train-HMD-VR group, a greater proportion of participants performed worse on the Transfer testing block compared to the Acquired Skill testing block (Fig. 4a, left). Conversely, in the Train-Screen group, a greater proportion of participants performed better on the Transfer testing block compared to the Leaned Skill testing block (Fig. 4a, right). To examine group and individual differences in transfer for each group, we first calculated the amount of motor skill transfer for each individual. To do this, we took the difference in motor skill between the Transfer testing block and the Acquired Skill testing block for each individual. At the group level, we compared the average motor skill transfer between the two groups and found a significant difference (t(61.5) = 3.75, *p* = 0.0004; Fig. 4b), where the motor skill transfer to a computer screen in the Train-HMD-VR group (M = − 0.31, SD = 0.67) was significantly lower that the motor skill transfer to HMD-VR in the Train-Screen group (M = 0.41, SD = 0.94). This suggests that the type of training environment during motor skill acquisition may affect the overall transfer of the motor skills to another environment; specifically, training in an HMD-VR environment may not transfer to a conventional environment. However, as seen in Fig. 4a, not all participants had similar transfer, suggesting that individual differences may predict the transfer of motor skill acquisition between environments.

### Predicting motor skill transfer

Given the interindividual variability of motor skill transfer (Fig. 4a), we were interested in whether any self-reported measurements collected (i.e., realism, possibility to act, quality of interface, possibility to examine, self-evaluation of performance, age, gender, video game use, and previous HMD-VR experience) could predict the motor skill transfer in each group. Using lasso with cross-validation to select the penalty term lambda, we performed variable selection to examine which of the nine variables most strongly predicted individual motor skill transfer (see *Analyses*). Additionally, we examined variables individually in each group with a univariate linear regression model for exploratory purposes.

#### Predicting HMD-VR motor skill transfer to a computer screen

For the Train-HMD-VR group, the resulting multivariate linear regression model retained four variables (Table 1) and explained 25.5% of the variance but was not statistically significant (F(4,30) = 2.57, R^2^ = 0.255, *p* = 0.0580). The model contained two presence variables predicting the motor skill transfer: positively correlated possibility to act and negatively correlated self-evaluation of performance. Multicollinearity was not an issue as the VIF for each variable was < 3.3.

**Table 1:** Results from a multivariate regression model for the Train-HMD-VR group

Predictor	Estimate	Std. Error	t-value	*p*-value
(Intercept)	−0.7396	0.7741	−0.9555	0.3470
Possibility to Act	0.0835	0.0382	2.1880	0.0366*
Self-Evaluation of Performance	−0.1515	0.0716	−2.1170	0.0426*
Video Game Play = Yes	0.1138	0.2380	0.4779	0.6362
Previous HMD-VR Experience = Yes	0.2836	0.2284	1.2420	0.2240

Possibility to act and self-evaluation of performance significantly predicted the amount of transfer from HMD-VR to a computer screen. *p* < 0.05^*^

**Fig. 5:**
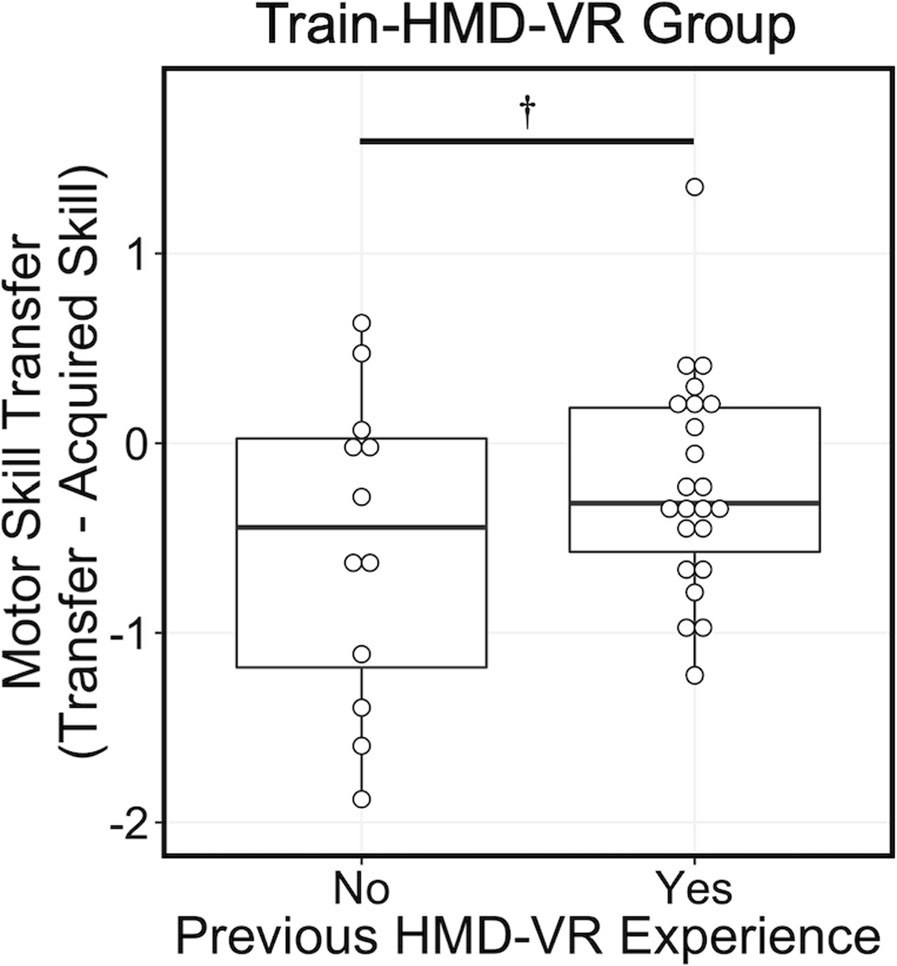
Train-HMD-VR Group: Individuals with previous HMD-VR experience had higher motor skill transfer to the screen compared to individuals who had never tried HMD-VR (F(1,33) = 2.90, R^2^ = 0.081, *p* = 0.0982). *p* < 0.1^†^

We did not find any significant results in examining variables individually in the univariate linear regression models (Supplementary Table 3). However, there was non-significant evidence of a difference in motor skill transfer in reported previous HMD-VR experience (F(1,33) = 2.90, R^2^ = 0.081, *p* = 0.0982) where individuals with previous HMD-VR experience had higher motor skill transfer (M = − 0.19, SD = 0.69) compared to individuals who had never tried HMD-VR (M = − 0.58, SD = 0.57; Fig. 5). Although these results are weak, they provide a preliminary suggestion that individual characteristics in these areas may explain why a reduction in motor skill may occur during HMD-VR transfer to a conventional environment. However, further research is needed to confirm these findings in a larger sample and with multiple tasks.

#### Predicting computer screen motor skill transfer to HMD-VR

For the Train-Screen group, the resulting multivariate linear regression model retained all nine variables (Table 2) and explained 59.7% of the variance (F (10,24) = 3.55, R^2^ = 0.597, *p* = 0.0053) with quality of interface, gender, age, and video game use significantly predicting the motor skill transfer, suggesting that the combination of these variables may be important for predicting computer screen motor skill transfer to HMD-VR. Multicollinearity was not an issue as the VIF for each variable was < 3.3.

**Table 2:** Results from multivariate regression model for Train-Screen

Predictor	Estimate	Std. Error	t-value	p-value
(Intercept)	1.205	1.191	1.012	0.3218
Realism	−0.04472	0.02562	−1.745	0.09372
Possibility to Act	−0.06161	0.05833	−1.056	0.3014
Quality of Interface	0.1027	0.0493	2.084	0.04798*
Possibility to Examine	0.07857	0.04586	1.713	0.09958
Self-evaluation of Performance	0.1428	0.08006	1.784	0.08715
Age	−0.0543	0.02388	−2.274	0.03222*
Gender = Male	0.9471	0.3572	2.652	0.01396*
Gender = Other	−0.1832	0.7773	−0.2357	0.8157
Video Game Use = Yes	−0.8933	0.2903	−3.077	0.005164**
Previous HMD-VR Experience = Yes	−0.2622	0.2848	−0.9208	0.3663

Quality of interface, gender, age, and video game use significantly predicted the amount of transfer from a computer screen to HMD-VR. *p* < 0.05^*^, *p* < 0.01^**^

**Fig. 6:**
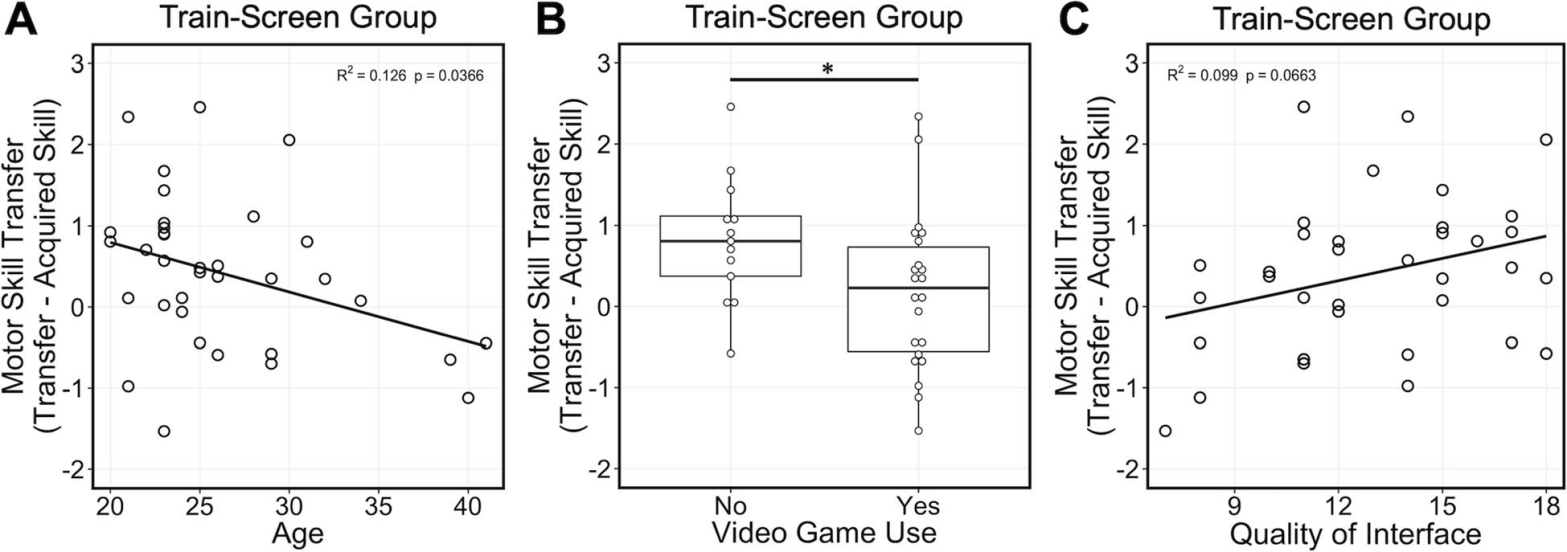
**Train-Screen Group: a** Younger age was significantly related to increased screen-based motor skill transfer to HMD-VR (F(1,33) = 4.75, R^2^ = 0.126, *p* = 0.0366). **b** Individuals who did not play video games had overall higher motor skill transfer to HMD-VR than individuals who played video games (F(1,33) = 4.15, R^2^ = 0.112, *p* = 0.0498). **c** Higher reports on the quality of the interface during training on a computer screen was related to increased computer screen motor skill transfer to HMD-VR; however, this result was non-significantly correlated (F(1,33) = 3.61, R^2^ = 0.099, *p* = 0.0663). *p* < 0.05^*^

Examining variables individually in the univariate linear regression models, we found significant results for age and video game use. Age was negatively correlated with motor skill transfer (F(1,33) = 4.75, R^2^ = 0.126, *p* = 0.0366; Fig. 6a), suggesting that younger age may facilitate transfer of the acquired motor skill to an HMD-VR environment. Additionally, there was significant evidence of a difference in motor skill transfer in reported video game use (F(1,33) = 4.15, R^2^ = 0.112, *p* = 0.0498; Fig. 6b) where individuals who did not play video games had overall higher motor skill transfer (M = 0.82, SD = 0.78) compared to individuals who played video games (M = 0.17, SD = 0.96). Furthermore, we found a non-significant positive trend between the quality of interface and motor skill transfer (F(1,33) = 3.61, R^2^ = 0.099, *p* = 0.0663; Fig. 6c), suggesting that this presence variable may be important in predicting computer screen motor skill transfer to HMD-VR; however, this should be further examined. Univariate linear regression results for Train-Screen can be found in Supplementary Table 4.

## Discussion

In this study, we examined motor skill transfer from an HMD-VR environment to a conventional environment (i.e., computer screen), and vice-versa. First, we confirmed that motor skill acquisition occurs in both HMD-VR and conventional screen environments and demonstrated that acquisition occurs at a similar rate in both environments, suggesting that task difficulty was not different between the environments. We then demonstrated that while motor skill transfer occurs after training in either environment, there are individual differences in the amount of motor skill that transferred.

In examining whether motor skills acquired during training in HMD-VR transferred to a conventional screen environment, we found a significant decrease in motor skill performance as a result of the transfer. To see if this decrease in motor skill transfer could be explained, we examined whether individual differences in five presence themes (realism, possibility to act, quality of interface, possibility to examine, self-evaluation of performance), age, gender, video game use, and previous HMD-VR experience could be used as predictors. We found trending but nonsignificant evidence that a combination of two presence themes, positively correlated possibility to act and negatively correlated self-evaluation of performance, best predicted this observed decrease in motor skill. Additionally, we found trending evidence that previous experience using HMD-VR independently may predict the decrease in the motor skill transfer. Overall, these results suggest that while the motor skills acquired in HMD-VR may not transfer to a conventional environment, the factors mentioned could mitigate this decrease.

We also examined whether motor skills acquired during training on a conventional screen environment transferred to HMD-VR. We found that motor skills learned in a conventional screen environment transfer to HMD-VR; however, not only do the motor skills transfer, but performances seem to improve in the novel HMD-VR environment. We found that the combination of the quality of interface, gender, age, and video game use best predicted this motor skill transfer. Additionally, we found evidence that age and video game use independently may predict the increase in motor skill transfer between computer screen and HMD-VR. This supports previous findings that age and video game use affect acquisition and transfer in non-immersive virtual environments [44, 45]. We also found trending evidence that the quality of interface independently may predict the increase in motor skill transfer between a computer screen and HMD-VR, further supporting the involvement of presence in the transfer of motor skill. These predictors may be useful to consider in cases when a HMD-VR rehabilitation intervention is introduced after motor skills have already been acquired in the real world.

Our work adds to the limited knowledge of personal factors that could potentially drive motor acquisition in HMD-VR and the transfer of motor skill to other environments. While other studies have identified potential mechanisms for HMD-VR transfer by examining existing literature [7], there is inconclusive evidence for why motor skill acquisition in HMD-VR and transfer to other environments may be more effective for some individuals compared to others. The two presence themes identified support previous findings that levels of presence relate to motor performance in an HMD-VR enviorment [46] and extend these findings to the transfer of motor skill acquisition. Additionally, previous experience with the training device, which is HMD-VR in the present case, support findings that the transfer of spatial knowledge is influenced by previous experience with the environment [31]. Increased exposure to HMD-VR may decrease the novelty, and subsequent attention evoked during the task, which may decrease motor performance. Future studies should examine whether individuals with more HMD-VR experience have greater motor skill transfer to the real world.

In addition to the personal factors that we have examined in this study, there are undoubtably more mechanisms that could either drive or predict HMD-VR motor skill transfer, and this should be further explored. Future studies should also consider other personal factors such as participants’ immersive tendencies, the likelihood that an individual will feel immersed in a new environment [47] as well as avatar embodiment, if applicable [48]. In addition to personal factors, task-related factors likely contribute to differences in motor skill acquisition and transfer from HMD-VR to conventional environments, and vice versa. Previous findings have suggested that fidelity and dimensionality influences the transfer of motor skills from HMD-VR environments [29]. In the current study, a possible explanation for the decrease in performance on a computer screen could be that the visual representation of the HMD-VR environment did not reflect what individuals expected and therefore, motor skill performance was not maintained with transfer. Future studies should consider examining the level of fidelity and dimensionally in HMD-VR needed to optimize motor skill transfer to the real world, and vice versa. Motor skill transfer has also been shown to be influenced by other task-related factors such as task variability, engagement, and feedback [49, 50]. In the current study, the increase in performance in HMD-VR could be a result of an increase in attention or engagement after transfer from the computer screen. Future studies should also examine how these task-related factors influence HMD-VR motor transfer to the real world, and vice versa.

It has also been suggested that HMD-VR may require additional cognitive resources and that additional information and stimuli must be processed in order to solve tasks in virtual reality [51]. One study found that the motor skills acquired in HMD-VR through the reliance of spatial cognitive capabilities did not transfer to the same task in the real world [25]. Our own previous work has shown that visuomotor adaptation in HMD-VR requires a greater reliance on cognitive strategies than performing the same task on a computer screen [19]. Taken together, this suggests that the decrease in motor skill transfer observed when moving to a conventional screen environment could also be due to less engagement of the cognitive processes used when in HMD-VR. The utilization of these cognitive processes during performance in either environment could be influenced by any of the personal or task-related factors described. Future work should examine whether specific cognitive processes have a role in HMD-VR motor skill transfer to the real world.

One limitation of this study was the use of a computer screen as the transfer condition from HMD-VR. Although this was purposefully designed to provide the most well-controlled and subtle differences between HMD-VR and the conventional environment, and previous studies have reported significant differences between HMD-VR and computer screen environments [19, 52–54], future work should examine whether presence, gender, age, video game use, or previous HMD-VR experience has an effect on HMD-VR motor skill transfer to more dynamic, real world physical applications (e.g., throwing a ball in HMD-VR versus throwing a ball in real life). Future research should also look to see if the identified factors apply to different clinical populations and examine whether mechanisms such as functional independence or cognitive status could predict success of HMD-VR rehabilitation interventions [55, 56]. Another limitation was that our definition of motor skill transfer reflects the transfer of motor skill acquisition rather than motor skill learning. Experimental designs of motor skill learning typically examine transfer after a retention interval and compare transfer performance to baseline performance in the transfer context [22]. Future studies should examine whether the personal factors identified here are also predictors for this type of experimental design. Lastly, the use of a subjective questionnaire to measure presence is also a limitation; future work should use alternative objective measures, such as physiological responses, in addition [57]. Overall, despite these limitations, we believe that the work presented in this study provides an initial examination into the transfer of motor skills between HMD-VR and conventional screen environments as well as insight into the factors that may mediate this transfer.

## Conclusion

Both HMD-VR and conventional screen environments resulted in the acquisition of a motor skill at a similar rate, as well as transfer to a different environment. However, motor skill performance decreased when transferring from HMD-VR to a conventional screen environment, while motor skill performance increased when transferring from a conventional screen environment to HMD-VR. Furthermore, themes of presence, gender, age, and video game use significantly predicted the motor skill transfer in individuals training on the screen, while themes of presence and previous HMD-VR experience were loosely related to the motor skill transfer in individuals training in HMD-VR. As HMD-VR becomes an increasingly popular medium for motor learning and rehabilitation applications, it is important to understand how to optimize interventions to ensure the complete transfer of motor skills to the target environment. Future studies should examine individual differences in other personal factors and in task-related factors.

## Supplementary information

**Additional file 1: Table S1.** Differences in simulator sickness level between Train-HMD-VR and Train-Screen. **Table S2.** Differences in themes of presence (top) and other self-reported measures (bottom) between Train-HMD-VR and Train-Screen. **Table S3.** Train-HMD-VR results from univariate analysis of predicting HMD-VR motor skill transfer to a computer screen. **Table S4.** Train-Screen results from univariate analysis of predicting computer screen motor skill transfer to HMD-VR.

## Data Availability

The data used in the current study are available from the corresponding author on reasonable request.

## Abbreviations

HMD-VR: virtual reality viewed using a head-mounted display
SVIPT: Sequential Visual Isometric Pinch Task
MSE: mean square error
VIF: variance inflation factor
