# Supplementary material for: Transfer of motor skill between virtual reality viewed using a head-mounted display and conventional screen environments
Source: J Neuroeng Rehabil. 2020 Apr 10;17:48. doi: 10.1186/s12984-020-00678-2 (PMC7149857; doi:10.1186/s12984-020-00678-2)
Supplement: Supplementary file 1 — Additional file 1: Table S1. Differences in simulator sickness level between Train-HMD-VR and Train-Screen. Table S2. Differences in themes of presence (top) and other self-reported measures (bottom) between Train-HMD-VR and Train-Screen. Table S3. Train-HMD-VR results from univariate analysis of predicting HMD-VR motor skill transfer to a computer screen. Table S4. Train-Screen results from univariate analysis of predicting computer screen motor skill transfer to HMD-VR. [file 12984_2020_678_MOESM1_ESM.docx]

**Supplementary Table 1. Differences in simulator sickness level between Train-HMD-VR and Train-Screen.**

|  | **U** | **n_1_ = n_2_** | **p-value** | **Train-HMD-VR** | **Train-Screen** |
| --- | --- | --- | --- | --- | --- |
| **Nausea** | 542 | 35 | 0.3741 | M = 2.18, SD = 2.18 | M = 4.63, SD = 13.16 |
| **Ocolomotor Reactions** | 675 | 35 | 0.4586 | M = 12.56, SD = 16.84 | M = 8.45, SD = 17.52 |
| **Disorientation** | 560 | 35 | 0.4881 | M = 7.16, SD = 18.91 | M = 6.76, SD = 16.67 |
| **Overall Simulator Sickness** | 609 | 35 | 0.9715 | M = 8.98, SD = 15.77 | M = 7.80, SD = 16.31 |

There were no significant differences in levels of nausea, oculomotor reactions, disorientation, and overall simulator sickness between groups.

**Supplementary Table 2. Differences in themes of presence (top) and other self-reported measures (bottom) between Train-HMD-VR and Train-Screen.**

|  | **t-value** | **df** | **p-value** | **Train-HMD-VR** | **Train-Screen** |
| --- | --- | --- | --- | --- | --- |
| **Realism** | 1.03 | 68.0 | 0.3082 | M = 34.86, SD = 7.31 | M = 33.06, SD = 7.36 |
| **Possibility to Act** | -0.28 | 67.3 | 0.7772 | M = 22.20, SD = 3.53 | M = 22.43, SD = 3.19 |
| **Quality of Interface** | 0.43 | 67.9 | 0.6655 | M = 13.34, SD = 3.38 | M = 13.00, SD = 3.23 |
| **Possibility to Examine** | 0.77 | 60.0 | 0.4430 | M = 15.00, SD = 2.59 | M = 14.40, SD = 3.80 |
| **Self-Evaluation of Performance** | -0.20 | 68.0 | 0.8435 | M = 11.20, SD = 1.81 | M = 11.29, SD = 1.81 |
| **Age** | -0.84 | 61.0 | 0.4069 | M = 25.34, SD = 3.84 | M = 26.29, SD = 5.46 |
|  |  |  |  |  |  |
|  | **χ^2^** | **df** | **p-value** | **Train-HMD-VR** | **Train-Screen** |
| **Gender** | 0.47 | 2 | 0.7919 | 25 female, 10 male | 18 female, 6 male,  1 other |
| **Video Game Use** | 2.27 | 1 | 0.1322 | 23 Yes, 12 No | 23 Yes, 13 No |
| **Previous HMD-VR Experience** | 0.04 | 1 | 0.8351 | 23 Yes, 12 No | 24 Yes, 11 No |

There were no significant differences in realism, possibility to act, quality of interface, possibility to examine, self-evaluation of performance, age, gender, video game use, and previous HMD-VR experience between groups.

**Supplementary Table 3. Train-HMD-VR results from univariate analysis of predicting HMD-VR motor skill transfer to a computer screen.**

| **Predictor** | **Estimate** | **Std. Error** | **t-value** | **p-value** |
| --- | --- | --- | --- | --- |
| **(Intercept)** | -0.3270 | 0.5700 | -0.5737 | 0.5700 |
| **Realism** | 0.0002 | 0.0160 | 0.0094 | 0.9926 |
|  |  |  |  |  |
| **(Intercept)** | -1.3640 | 0.7226 | -1.8880 | 0.0679 |
| **Possibility to Act** | 0.0470 | 0.0322 | 1.4600 | 0.1537 |
|  |  |  |  |  |
| **(Intercept)** | -0.1755 | 0.4761 | -0.3687 | 0.7147 |
| **Quality of Interface** | -0.0110 | 0.0347 | -0.3166 | 0.7535 |
|  |  |  |  |  |
| **(Intercept)** | -0.7376 | 0.6842 | -1.0780 | 0.2888 |
| **Possibility to Examine** | 0.0278 | 0.0450 | 0.6164 | 0.5418 |
|  |  |  |  |  |
| **(Intercept)** | 0.4028 | 0.7219 | 0.5580 | 0.5806 |
| **Self-Evaluation of Performance** | -0.0647 | 0.0637 | -1.0170 | 0.3168 |
|  |  |  |  |  |
| **(Intercept)** | 0.0408 | 0.7784 | 0.0524 | 0.9585 |
| **Age** | -0.0143 | 0.0304 | -0.4710 | 0.6408 |
|  |  |  |  |  |
| **(Intercept)** | -0.3772 | 0.1354 | -2.7860 | 0.0088 |
| **Gender = Male** | 0.1939 | 0.2533 | 0.7654 | 0.4495 |
|  |  |  |  |  |
| **(Intercept)** | -0.5319 | 0.1919 | -2.7720 | 0.0091 |
| **Video Game Use = Yes** | 0.3197 | 0.2367 | 1.3510 | 0.1860 |
|  |  |  |  |  |
| **(Intercept)** | -0.5826 | 0.189 | -3.0820 | 0.0041 |
| **Previous HMD-VR Experience = Yes** | 0.3968 | 0.2331 | 1.7020 | **0.0982^†^** |

There was non-significant evidence of a difference in motor skill transfer in reported previous HMD-VR experience. p < 0.1^†^.

**Supplementary Table 4. Train-Screen results from univariate analysis of predicting computer screen motor skill transfer to HMD-VR.**

| **Predictor** | **Estimate** | **Std. Error** | **t-value** | **p-value** |
| --- | --- | --- | --- | --- |
| **(Intercept)** | 0.2554 | 0.7540 | 0.3388 | 0.7369 |
| **Realism** | 0.0047 | 0.0223 | 0.2121 | 0.8333 |
|  |  |  |  |  |
| **(Intercept)** | 0.4707 | 1.1630 | 0.4047 | 0.6883 |
| **Possibility to Act** | -0.0026 | 0.0514 | -0.0513 | 0.9594 |
|  |  |  |  |  |
| **(Intercept)** | -0.7799 | 0.6458 | -1.2080 | 0.2358 |
| **Quality of Interface** | 0.0917 | 0.0483 | 1.8990 | **0.0663^†^** |
|  |  |  |  |  |
| **(Intercept)** | -0.2126 | 0.6326 | -0.3361 | 0.7389 |
| **Possibility to Examine** | 0.0434 | 0.0425 | 1.0200 | 0.3153 |
|  |  |  |  |  |
| **(Intercept)** | -0.8375 | 1.0130 | -0.8269 | 0.4142 |
| **Self-Evaluation of Performance** | 0.1107 | 0.0886 | 1.2490 | 0.2206 |
|  |  |  |  |  |
| **(Intercept)** | 2.0190 | 0.7530 | 2.6810 | 0.0114 |
| **Age** | -0.0611 | 0.0281 | -2.1780 | **0.0366^*^** |
|  |  |  |  |  |
| **(Intercept)** | 0.3288 | 0.1740 | 1.8890 | 0.0680 |
| **Gender = Male** | 0.6367 | 0.4143 | 1.5370 | 0.1342 |
| **Gender = Other** | -0.9192 | 0.9372 | -0.9807 | 0.3341 |
|  |  |  |  |  |
| **(Intercept)** | 0.8152 | 0.2500 | 3.2610 | 0.0026 |
| **Video Game Use = Yes** | -0.6420 | 0.3153 | -2.0360 | **0.0498^*^** |
|  |  |  |  |  |
| **(Intercept)** | 0.4776 | 0.2880 | 1.6590 | 0.1067 |
| **Previous HMD-VR Experience = Yes** | -0.0963 | 0.3478 | -0.2767 | 0.7837 |

Significant results were found with age and video use. Additionally, a non-significant positive trend between the quality of interface and motor skill transfer was found. p < 0.05^*^, p < 0.1^†^.
